# A Randomized Double-Blind Placebo-Controlled Trial of a Botanical Formulation for Symptom Management in Male Climacteric Syndrome

**DOI:** 10.3390/medicina62071334

**Published:** 2026-07-10

**Authors:** Da-In Choi, Jang Hee Hong, Gyuok Lee, Jaeyong Kim, Chul-Yung Choi

**Affiliations:** 1 Institute of Well-Aging Medicare & Chosun University G-LAMP Project Group, Chosun University, Gwangju 61452, Republic of Korea; 2 Department of Pharmacology, College of Medicine, Chungnam National University, Daejeon 34134, Republic of Korea; 3 Clinical Trials Center, Chungnam National University Hospital, Daejeon 35015, Republic of Korea; 4 Jeonnam Bioindustry, Jangheung-gun 57000, Republic of Korea; 5 Department of Biomedical Science, Chosun University, Gwangju 61452, Republic of Korea; 6 Department of Integrative Biological Sciences & BK21 FOUR Educational Research Group for Age-Associated Disorder Control Technology, Chosun University, Gwangju 61452, Republic of Korea

**Keywords:** male climacteric syndrome, placebo response, testosterone variability, botanical extract, non-hormonal therapy, *Elaeagnus multiflora*, *Cynanchum wilfordii*, randomized controlled trial

## Abstract

*Background/Objectives:* Male climacteric syndrome (MCS) affects quality of life through psychological, somatic, sexual, and endocrine-related symptoms. This study evaluated whether a botanical formulation containing *Elaeagnus multiflora* fruit and *Cynanchum wilfordii* extracts (EMF_CWW) improved MCS-related symptoms compared with placebo. *Methods:* We conducted a 12-week, randomized, double-blind, placebo-controlled trial (Clinical Research Information Service registration: KCT0010695) in 70 men aged 45–75 years with MCS. Participants received EMF_CWW (1500 mg/day) or placebo. The primary outcome was the change in Aging Males’ Symptoms (AMS) total score from baseline to Week 12. Secondary outcomes included AMS subdomains, Androgen Deficiency in Aging Males (ADAM) positivity, hormonal parameters, lipid profile, and safety. Efficacy analyses were conducted in the per-protocol population (*n* = 64), and safety analyses were conducted in the safety population (*n* = 70). *Results:* EMF_CWW did not demonstrate superiority over placebo for the primary outcome. AMS total score decreased in both groups (treatment: −14.2 ± 12.5; placebo: −15.5 ± 12.3), with no significant between-group difference (*p* = 0.6726). AMS subdomains and ADAM positivity also improved in both groups without significant between-group differences. Free testosterone increased non-significantly in the treatment group (+0.8 ± 2.4 pg/mL; *p* = 0.0727) and significantly in the placebo group (+1.3 ± 2.6 pg/mL; *p* = 0.0076), with no between-group difference (*p* = 0.8720). No treatment-related serious adverse events were observed. *Conclusions:* EMF_CWW did not demonstrate efficacy superior to placebo for improving MCS-related symptoms or hormonal outcomes. The improvements observed in both groups highlight placebo responsiveness, regression to the mean, and symptom variability in MCS trials. No apparent short-term safety signal was observed; however, long-term safety cannot be concluded. Larger trials with standardized repeated morning testosterone measurements are required.

## 1. Introduction

Aging in men is accompanied by a gradual decline in total and free testosterone and by a constellation of sexual, somatic, and psychological symptoms commonly grouped under the term male climacteric syndrome (MCS) [1]. This decline is slow and heterogeneous, and symptom burden correlates only weakly with circulating testosterone [1,2]. The European Male Aging Study (EMAS) identified only a small subset of men in whom specific symptoms co-occurred with unequivocally low testosterone, indicating that most symptomatic aging men do not meet a strict biochemical definition of hypogonadism [2]. Current guidelines accordingly reserve a diagnosis of hypogonadism for men with consistent symptoms together with repeatedly low morning testosterone, while recognizing that symptomatic men with low-normal testosterone constitute a distinct and clinically common group [3,4].

For men with confirmed hypogonadism, testosterone replacement therapy (TRT) can improve sexual function, mood, and energy [5,6,7]. Its use has historically been tempered by concerns regarding cardiovascular events [8,9,10,11,12]; more recent evidence has partly addressed this question, as the TRAVERSE trial reported that TRT was non-inferior to placebo for major adverse cardiovascular events in men with hypogonadism and high cardiovascular risk, albeit with a higher incidence of atrial fibrillation [13]. Additional safety considerations include erythrocytosis [14] and prostate-related adverse effects [15]. Importantly, TRT is not indicated for the many symptomatic aging men who have low-normal rather than unequivocally low testosterone, and its risk-benefit profile cannot itself justify the use of unproven non-hormonal alternatives. These factors have nonetheless sustained interest in non-hormonal options, which require their own rigorous, controlled evaluation.

*Elaeagnus multiflora fruit* and *Cynanchum wilfordii* have long been used in East Asian traditional medicine for vitality and aging-related complaints [16,17]. Preclinical studies of the individual extracts have reported antioxidant activity and androgen-related effects [16,18], and our group previously showed that a mixed *Elaeagnus multiflora* and *Cynanchum wilfordii* formulation increased testosterone in TM3 Leydig cells and in aging male rats [17]. Whether these preclinical signals translate into symptomatic or hormonal benefit in symptomatic aging men, however, has not been established in rigorous placebo-controlled trials.

Two factors complicate the interpretation of trials in this setting and are often insufficiently addressed. First, placebo responsiveness is pronounced for the subjective, psychosomatic symptoms that dominate MCS, so that symptom scores frequently improve substantially in placebo arms. Second, endogenous testosterone is highly variable: because participants are typically enrolled on the basis of low screening values, regression to the mean may produce apparent biochemical improvement independent of any intervention, and unstandardized sampling adds further measurement-related variability. Accounting for these factors is essential when evaluating any non-hormonal intervention in symptomatic aging men [19].

Against this background, we conducted a 12-week, randomized, double-blind, placebo-controlled trial of a standardized non-hormonal botanical formulation (EMF_CWW) in symptomatic men aged 45–75 years. The primary objective was to determine whether EMF_CWW reduced the Aging Males’ Symptoms (AMS) total score more than placebo; secondary objectives addressed AMS subdomains, Androgen Deficiency in Aging Males (ADAM) positivity, hormonal parameters, lipid profile, and safety. Recognizing the interpretive challenges outlined above, we additionally examined symptom trajectories, placebo responsiveness, and testosterone variability, with the aim of informing the design and interpretation of future trials in this population.

## 2. Materials and Methods

### 2.1. Study Design and Ethics

This 12-week, randomized, double-blind, placebo-controlled trial was conducted at Chungnam National University Hospital, Daejeon, Republic of Korea, from 27 August 2018, to 14 August 2019. The protocol was approved by the Chungnam National University Hospital Institutional Review Board on 1 June 2018 before participant enrollment (IRB No. CNUH IRB 2018-05-026), and the study was retrospectively registered with the Clinical Research Information Service on 1 July 2025 (CRIS KCT0010695). The study was registered retrospectively to maintain a transparent public record after the long interval between study completion and publication. All participants provided written informed consent. The study complied with Good Clinical Practice guidelines and the Declaration of Helsinki. Lifestyle variables such as sleep duration, stress level, alcohol intake, and exercise frequency were not restricted; however, participants were instructed to avoid initiating new health-related routines during the study period to minimize behavioral confounding.

### 2.2. Participants

Men aged 45–75 years with MCS were recruited through hospital outpatient clinics and community advertisements. Inclusion criteria: (1) AMS total score ≥ 27; (2) positive ADAM questionnaire; (3) serum total testosterone ≤ 8.59 ng/mL; (4) ability to provide informed consent. Exclusion criteria: (1) prostate cancer or PSA > 4 ng/mL; (2) severe cardiovascular disease; (3) uncontrolled diabetes or hypertension; (4) liver or kidney dysfunction; (5) current TRT or herbal supplements; (6) psychiatric disorders; (7) known allergy to study ingredients.

### 2.3. Randomization and Blinding

Participants were randomized in a 1:1 ratio to receive either EMF_CWW or placebo according to a predefined randomization schedule. This double-blind, parallel-group trial maintained allocation concealment through an independent dispensing pharmacist not involved in outcome assessment. Investigators, clinical staff, and participants remained blinded until database lock.

### 2.4. Interventions

Participants were randomized to receive either EMF_CWW or placebo for 12 weeks. The EMF_CWW capsule contained 500 mg per capsule, consisting of *Cynanchum wilfordii* extract (245 mg), *Elaeagnus multiflora* fruit extract (105 mg), and crystalline cellulose (150 mg). Participants were instructed to take three capsules once daily after dinner, corresponding to a total daily dose of 1500 mg.

The preparation method, mixing ratio, and preclinical rationale for the *Elaeagnus multiflora* fruit and *Cynanchum wilfordii* extract mixture have been described in a previous preclinical study by our research group. In that study, the EMF:CWW mixture at a ratio of 3:7 was selected based on testosterone production in TM3 Leydig cells and was further evaluated in aging male rats. The EMCW formulation used in the preclinical study was reported to be the same as that used in the present clinical trial [17].

For additional characterization of the herbal extracts, HPLC fingerprint analysis was performed for the individual water extracts and the combined extract used for the clinical product. Detailed HPLC instrumentation, analytical conditions, marker information, and chromatographic profiles are provided in (Supplementary Document). The *Elaeagnus multiflora* fruit water extract showed a phenolic acid-type marker peak, whereas the *Cynanchum wilfordii* water extract showed a 4′-hydroxyacetophenone marker peak. In the combined extract prepared at a CWW:EMF ratio of 7:3, the 4′-hydroxyacetophenone peak was observed at approximately 15.3 min, and the phenolic acid-type marker peak was observed at approximately 28.1 min, without co-elution between the two marker regions. The extraction conditions, marker information, and HPLC chromatographic profiles are provided in (Supplementary Materials).

The placebo capsule contained crystalline cellulose, gardenia yellow pigment, gardenia blue pigment, magnesium stearate, silicon dioxide, and caramel coloring, and was designed to be identical in appearance to the EMF_CWW capsule. Participants in the placebo group also took three capsules once daily after dinner for 12 weeks.

According to the Clinical Study Report, the study product and placebo were packaged, labeled, and supplied to the study site through Jeonnam Bioindustry Natural Resources Research Center (Jangheung-gun, Jeollanam-do, Republic of Korea). Product labels included the study-use designation, product code or ingredient name, manufacturing number, expiration date, and storage conditions.

### 2.5. Outcome Measures

(1) Primary Outcome: Change in AMS total score from baseline to Week 12. The AMS is a validated 17-item questionnaire assessing psychological (5 items), somatic (7 items), and sexual (5 items) symptoms, scored from 1 to 5 for each item (total range: 17–85) [19].
(2) Secondary Outcomes: Secondary outcomes included changes in AMS subdomain scores, positivity on the Androgen Deficiency in Aging Males (ADAM) questionnaire [20], hormonal parameters (total testosterone, free testosterone, and SHBG), lipid profiles (total cholesterol, LDL, HDL, and triglycerides), and safety outcomes including adverse events, vital signs, and laboratory parameters.

### 2.6. Assessment Schedule

Participants attended four visits: screening, baseline, Week 6, and Week 12. Vital signs, physical examination, medication use, study product dispensing, compliance, and adverse events were assessed according to the study schedule. AMS and ADAM questionnaires were assessed at baseline and Week 12. Laboratory evaluations included hematology, blood chemistry, urinalysis, lipid parameters, prostate-specific antigen, and hormonal parameters.

Total testosterone, free testosterone, and sex hormone-binding globulin (SHBG) were assessed as hormonal parameters. Total testosterone was measured at screening and Week 12, whereas free testosterone and SHBG were measured at baseline and Week 12, according to the CSR schedule. Blood samples for lipid and hormonal analyses were collected after overnight fasting; however, the exact time of blood collection was not fixed across participants. The assay platform, analytical method, and intra- and inter-assay coefficients of variation were not specified in the CSR. Therefore, hormonal findings, particularly free testosterone changes, should be interpreted cautiously.

### 2.7. Statistical Analysis

All statistical analyses were originally planned and performed using SAS software (Version 9.4; SAS Institute Inc., Cary, NC, USA), as specified in the Clinical Study Report (CSR). Continuous variables are presented as mean ± standard deviation (SD), and categorical variables are presented as number (percentage).

Baseline demographic characteristics were summarized descriptively by treatment group. No formal significance testing was performed for baseline demographic variables, in accordance with CONSORT recommendations.

The per-protocol (PP) population was designated as the main efficacy analysis set according to the CSR analysis plan. The PP population included participants who completed the 12-week intervention, had no major protocol deviations, and demonstrated compliance of at least 80% with the assigned study product or placebo. The safety population included all randomized participants who received at least one dose of the study product or placebo.

The primary endpoint was the change in Aging Males’ Symptoms (AMS) total score from baseline to Week 12. Changes were calculated as Week 12 minus baseline. Within-group changes in continuous outcomes were analyzed using paired *t*-tests, and between-group comparisons of changes were performed using independent *t*-tests. For categorical outcomes, including ADAM positivity, within-group changes were assessed using McNemar’s test, and between-group comparisons were performed using the chi-square test or Fisher’s exact test, as appropriate. All statistical tests were two-sided, and *p* < 0.05 was considered statistically significant.

To address the potential influence of excluding non-completers from the PP population, a conservative intention-to-treat (ITT) sensitivity analysis was additionally performed for the primary and key secondary continuous outcomes. The ITT population included all randomized participants who received at least one dose of the study product or placebo (*n* = 70; treatment, *n* = 35; placebo, *n* = 35). Missing Week 12 values were imputed using baseline observation carried forward (BOCF), assuming no change from baseline for participants without evaluable Week 12 data. This analysis was conducted to assess whether the primary interpretation was robust to the use of the PP population as the main efficacy analysis set.

The target sample size was 70 randomized participants, with 35 participants per group, allowing for an anticipated dropout rate of approximately 20% to obtain at least 56 completers. This sample size was based on an assumed 10-point between-group difference in AMS total score, an SD of 15, 80% statistical power, and a two-sided significance level of 0.05.

## 3. Results

### 3.1. Participant Flow and Baseline Characteristics

**Figure 1:**
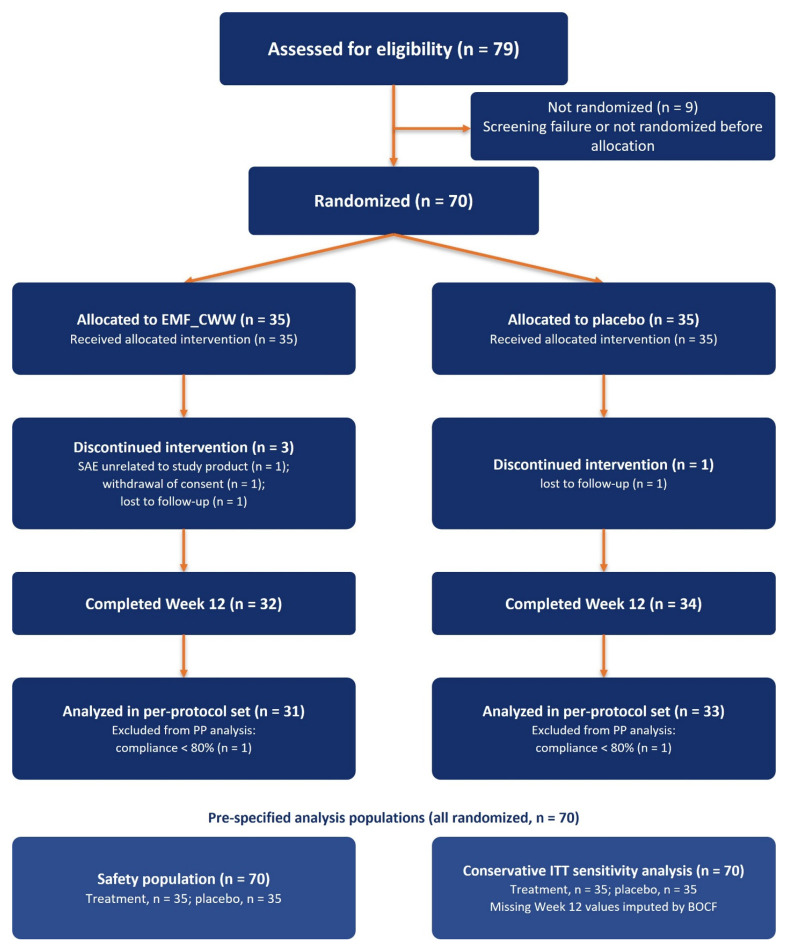
CONSORT flow diagram. A total of 79 men were assessed for eligibility, of whom 70 were randomized to receive EMF_CWW or placebo. The safety population included all randomized participants who received the study product or placebo (*n* = 70). Four participants discontinued the intervention before Week 12, and 66 participants completed the Week 12 visit. The per-protocol population included 64 participants after excluding two participants with compliance below 80%. A conservative intention-to-treat sensitivity analysis was additionally performed using baseline observation carried forward.

A total of 79 men were screened, of whom 70 were randomized, with 35 assigned to the treatment group and 35 assigned to the placebo group. Six participants were excluded from the per-protocol (PP) population because of discontinuation (*n* = 4; including two participants lost to follow-up, one withdrawal of consent, and one serious adverse event unrelated to the study product) or compliance below 80% (*n* = 2). Accordingly, the PP population included 64 participants, with 31 in the treatment group and 33 in the placebo group. Participant disposition is summarized in the CONSORT flow diagram (Figure 1). Safety analyses were conducted in all randomized participants who received the study product or placebo (safety population, *n* = 70).

Baseline demographic characteristics of the PP population are summarized descriptively in Table 1. The treatment and placebo groups were generally similar in age, height, and weight. Baseline AMS total score and hormonal parameters are presented in the efficacy outcome table.

The overall study schedule, including screening procedures, outcome assessments, laboratory evaluations, and follow-up visits, is summarized in Supplementary Table S1.

### 3.2. Primary Outcome: AMS Total Score

**Figure 2:**
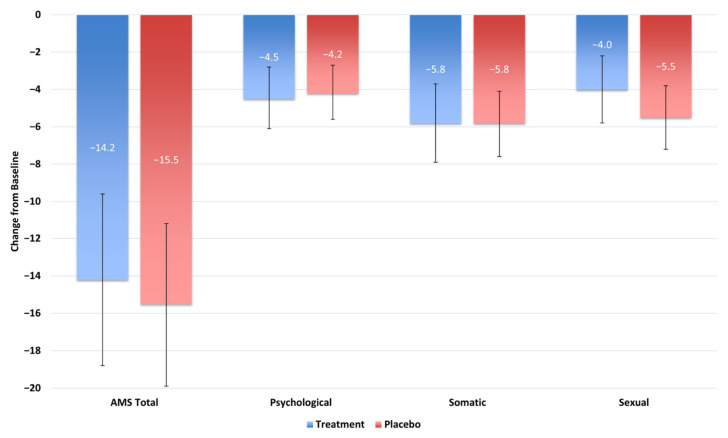
Changes in Aging Males’ Symptoms (AMS) scores from baseline to Week 12 in the treatment and placebo groups. Data are presented as mean changes from baseline with 95% confidence intervals. Negative values indicate symptom improvement. AMS domains include the total score and psychological, somatic, and sexual subdomain scores. Although both groups showed reductions in AMS scores, no statistically significant between-group differences were observed for the AMS total score or any subdomain.

The primary outcome was the change in AMS total score from baseline to Week 12. EMF_CWW did not demonstrate superiority over placebo for the primary outcome (Table 2, Figure 2). In the treatment group, AMS total score decreased from 45.0 ± 9.5 at baseline to 30.8 ± 11.1 at Week 12, corresponding to a mean change of −14.2 ± 12.5 points (95% CI: −18.8 to −9.6; *p* < 0.001). In the placebo group, AMS total score decreased from 43.7 ± 10.2 to 28.2 ± 11.0, corresponding to a mean change of −15.5 ± 12.3 points (95% CI: −19.9 to −11.2; *p* < 0.001). The between-group difference in change was not statistically significant (*p* = 0.6726), indicating that the improvement observed in the treatment group was not greater than that observed in the placebo group. A conservative ITT sensitivity analysis using BOCF showed the same overall interpretation as the PP analysis (Supplementary Table S2). AMS total score decreased in both groups, but EMF_CWW did not demonstrate superiority over placebo.

### 3.3. Secondary Outcomes

#### 3.3.1. AMS Subdomain Scores

AMS psychological, somatic, and sexual subdomain scores decreased from baseline to Week 12 in both groups; however, no statistically significant between-group differences were observed for any AMS subdomain (Table 2, Figure 2, and Supplementary Table S3).

- Psychological subdomain scores decreased from 12.5 ± 3.1 to 8.1 ± 3.9 in the treatment group (mean change: −4.5 ± 4.4; 95% CI: −6.1 to −2.8; *p* < 0.001) and from 12.0 ± 3.8 to 7.8 ± 3.6 in the placebo group (mean change: −4.2 ± 4.1; 95% CI: −5.6 to −2.7; *p* < 0.001). The between-group difference was not statistically significant (*p* = 0.8021).
- Somatic subdomain scores decreased from 18.4 ± 4.1 to 12.5 ± 4.9 in the treatment group (mean change: −5.8 ± 5.6; 95% CI: −7.9 to −3.7; *p* < 0.001) and from 17.1 ± 4.9 to 11.3 ± 4.4 in the placebo group (mean change: −5.8 ± 5.0; 95% CI: −7.6 to −4.1; *p* < 0.001). The between-group difference was not statistically significant (*p* = 0.9930).
- Sexual subdomain scores decreased from 14.2 ± 3.8 to 10.2 ± 4.0 in the treatment group (mean change: −4.0 ± 4.9; 95% CI: −5.8 to −2.2; *p* < 0.001) and from 14.6 ± 3.3 to 9.1 ± 3.5 in the placebo group (mean change: −5.5 ± 4.8; 95% CI: −7.2 to −3.8; *p* < 0.001). The between-group difference was not statistically significant (*p* = 0.1965).

Exploratory age-stratified analyses showed variable patterns of AMS score reduction across age categories (Supplementary Table S4). Because these analyses were post hoc and subgroup sizes were small, the findings should be interpreted descriptively. Overall, the age-stratified results did not provide evidence of a consistent treatment-specific benefit of EMF_CWW over placebo.

#### 3.3.2. ADAM Questionnaire

ADAM positivity decreased from baseline to Week 12 in both groups (Table 2). In the treatment group, 29 of 31 participants (93.5%) were ADAM-positive at baseline, decreasing to 15 of 31 participants (48.4%) at Week 12 (McNemar’s test, *p* < 0.001). In the placebo group, 32 of 33 participants (97.0%) were ADAM-positive at baseline, decreasing to 20 of 33 participants (60.6%) at Week 12 (McNemar’s test, *p* < 0.001). However, the between-group difference in ADAM positivity at Week 12 was not statistically significant (*p* = 0.3264), indicating that the observed reduction was not specific to EMF_CWW treatment. (Supplementary Table S5).

**Figure 3:**
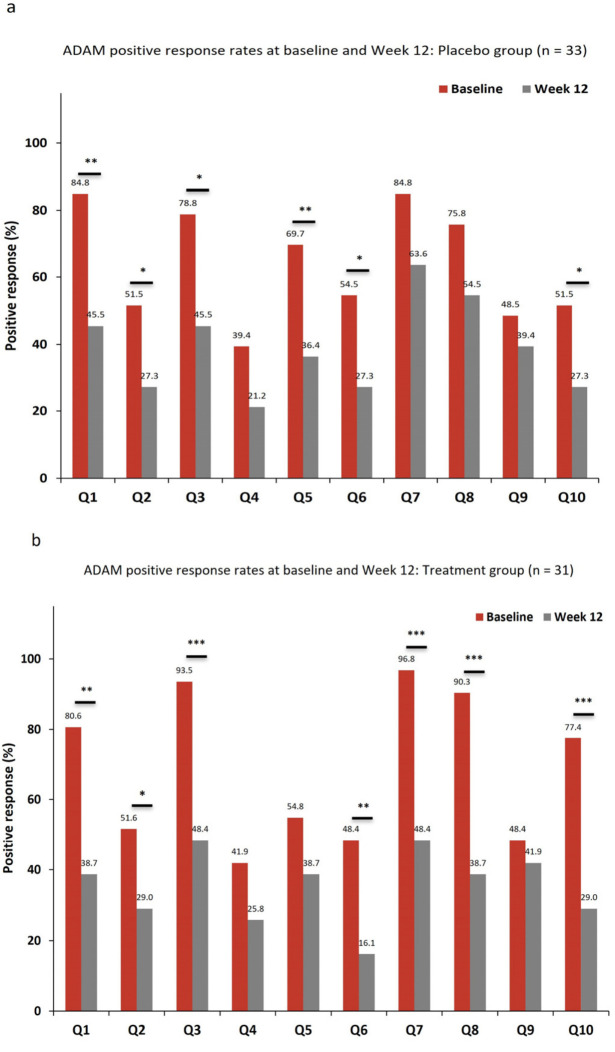
Item-level positive response rates for the Androgen Deficiency in Aging Males (ADAM) questionnaire at baseline and Week 12. (**a**) Placebo group (*n* = 33). (**b**) Treatment group (*n* = 31). Data are presented as the percentage of participants with a positive response to each ADAM item at baseline and Week 12. Q1, decreased libido; Q2, lack of energy; Q3, decreased strength or endurance; Q4, loss of height; Q5, decreased enjoyment of life; Q6, sadness or grumpiness; Q7, weaker erections; Q8, reduced ability to engage in sports; Q9, falling asleep after dinner; Q10, deterioration in work performance. Within-group changes from baseline to Week 12 were analyzed using McNemar’s exact test. * *p* < 0.05, ** *p* < 0.01, *** *p* < 0.001. Lower percentages at Week 12 indicate fewer positive ADAM symptoms. Item-level findings are descriptive and should be interpreted cautiously because no statistically significant between-group difference was observed for overall ADAM positivity.

Item-level ADAM positive response rates at baseline and Week 12 are shown descriptively in (Figure 3). Reductions in positive responses were observed across several ADAM items in both groups; however, these item-level patterns should be interpreted as exploratory because no statistically significant between-group difference was observed for overall ADAM positivity. Participant-level changes in individual ADAM questionnaire items are further illustrated in (Supplementary Figure S1), showing heterogeneous response patterns across participants in both treatment arms.

### 3.4. Hormonal Parameters

Hormonal parameters were evaluated as secondary exploratory outcomes (Table 2 and Supplementary Table S6). Overall, no statistically significant between-group differences were observed in total testosterone, free testosterone, or sex hormone-binding globulin (SHBG) after 12 weeks of intervention. Because hormonal sampling was not time standardized, these findings should be interpreted cautiously.

#### 3.4.1. Total Testosterone

In the treatment group, total testosterone changed from 4.2 ± 1.3 ng/mL at baseline to 4.0 ± 1.3 ng/mL at Week 12 (mean change: −0.2 ± 1.3 ng/mL; 95% CI: −0.7 to 0.3; *p* = 0.3902). In the placebo group, total testosterone changed from 4.5 ± 1.2 ng/mL to 4.6 ± 1.3 ng/mL (mean change: +0.1 ± 1.2 ng/mL; 95% CI: −0.3 to 0.5; *p* = 0.6783). The between-group difference was not statistically significant (*p* = 0.3497).

#### 3.4.2. Free Testosterone

In the treatment group, free testosterone increased from 7.7 ± 2.2 pg/mL to 8.6 ± 2.0 pg/mL, but this within-group change did not reach statistical significance (mean change: +0.8 ± 2.4 pg/mL; 95% CI: −0.1 to 1.7; *p* = 0.0727). In the placebo group, free testosterone increased from 7.9 ± 2.6 pg/mL to 9.2 ± 2.4 pg/mL (mean change: +1.3 ± 2.6 pg/mL; 95% CI: 0.4 to 2.2; *p* = 0.0076). However, the between-group difference was not statistically significant (*p* = 0.8720). Therefore, the observed free testosterone changes do not support a treatment-specific hormonal effect of EMF_CWW.

#### 3.4.3. SHBG

SHBG levels showed no statistically significant between-group difference. In the treatment group, SHBG changed from 44.2 ± 13.0 nmol/L to 45.9 ± 13.6 nmol/L (mean change: +1.7 ± 5.8 nmol/L; 95% CI: −0.4 to 3.8; *p* = 0.0889). In the placebo group, SHBG changed from 54.3 ± 17.8 nmol/L to 54.7 ± 18.6 nmol/L (mean change: +0.4 ± 7.3 nmol/L; 95% CI: −2.2 to 3.0; *p* = 0.7889). The between-group difference was not statistically significant (*p* = 0.3362).

### 3.5. Lipid Profile

Lipid parameters were assessed as secondary safety-related laboratory outcomes (Supplementary Table S7). No statistically significant between-group differences were observed for total cholesterol, LDL-cholesterol, HDL-cholesterol, or triglycerides during the 12-week intervention.

- Total cholesterol showed no statistically significant within-group change in either the treatment group (196.7 ± 42.1 to 195.8 ± 35.7 mg/dL; *p* = 0.8246) or the placebo group (191.3 ± 26.8 to 193.9 ± 23.1 mg/dL; *p* = 0.4744), with no significant between-group difference (*p* = 0.5076).
- LDL-cholesterol also remained statistically unchanged in both groups (treatment: 119.6 ± 36.2 to 120.5 ± 31.2 mg/dL; *p* = 0.7726; placebo: 112.6 ± 25.5 to 114.9 ± 22.4 mg/dL; *p* = 0.4831), with no significant between-group difference (*p* = 0.7657).
- HDL-cholesterol did not differ significantly over time in either group (treatment: 51.5 ± 12.4 to 50.0 ± 11.5 mg/dL; *p* = 0.2973; placebo: 56.1 ± 14.4 to 56.1 ± 14.6 mg/dL; *p* = 0.9538), and the between-group difference was not statistically significant (*p* = 0.4174).
- Triglyceride levels showed no statistically significant within-group change in either the treatment group (183.0 ± 99.5 to 176.1 ± 90.7 mg/dL; *p* = 0.6826) or the placebo group (178.7 ± 173.6 to 148.1 ± 128.7 mg/dL; *p* = 0.3431), with no significant between-group difference (*p* = 0.5148).

Overall, lipid parameters remained statistically stable over the 12-week study period. These findings suggest no apparent short-term unfavorable changes in lipid-related laboratory parameters; however, they should not be interpreted as evidence of long-term metabolic safety.

### 3.6. Safety

Safety analyses were conducted in the safety population, defined as all randomized participants who received at least one dose of the study product or placebo (*n* = 70; treatment, *n* = 35; placebo, *n* = 35). During the 12-week intervention, adverse events were reported in 2 of 35 participants (5.7%) in the treatment group and 6 of 35 participants (17.1%) in the placebo group (Fisher’s exact test, *p* = 0.2595) (Table 3). A total of 9 adverse event episodes were recorded, including 2 in the treatment group and 7 in the placebo group.

One serious adverse event occurred in the treatment group and was assessed by the investigator as unrelated to the study product. This event resolved without sequelae. No treatment-related serious adverse events were reported. Most adverse events were mild to moderate in severity, although severe events were reported in 1 participant in each group. All participants with adverse events recovered or resolved without sequelae.

Overall, no apparent short-term safety signal was observed during the 12-week study period. However, given the small sample size and limited follow-up duration, these findings support only short-term tolerability and should not be interpreted as evidence of long-term safety.

## 4. Discussion

### 4.1. Overall Interpretation of the Trial Findings

In this randomized, double-blind, placebo-controlled trial, EMF_CWW, a standardized non-hormonal formulation of *Elaeagnus multiflora* and *Cynanchum wilfordii*, was not superior to placebo in symptomatic aging men for the primary outcome or for any secondary outcome. AMS total and subdomain scores and ADAM positivity improved substantially in both arms with no between-group difference, and no treatment-specific hormonal effect was detected. The trial should therefore be read as a non-positive study whose data do not support an efficacy benefit of EMF_CWW beyond placebo. Over the 12-week period EMF_CWW was well tolerated; however, this short, modestly sized study was not designed to establish safety, and only short-term tolerability can be inferred.

### 4.2. Nonspecific Contributors to the Improvement Observed in Both Arms

The substantial within-arm improvement in the absence of any between-group difference is most parsimoniously explained by nonspecific factors common to both arms rather than by a specific action of EMF_CWW. These include the recognized placebo responsiveness of subjective symptoms in testosterone-deficiency and sexual-dysfunction research [21,22,23], regression to the mean in participants selected for elevated symptom scores, and behavioral change associated with trial participation. Because the randomized, placebo-controlled design already controls for these nonspecific influences, the appropriate conclusion is that EMF_CWW did not separate from placebo, not that a specific effect was masked.

### 4.3. Psychosomatic Characteristics of MCS Symptoms

The predominance of subjective, psychosomatic symptoms in MCS—fatigue, low mood, and reduced libido—plausibly underlies this responsiveness [23]. Regular contact, monitoring, and the expectation of benefit inherent in trial participation can improve such symptoms independently of any pharmacological effect [21,23].

### 4.4. Potential Influence of Lifestyle and Behavioral Changes

Enrollment in a clinical trial can itself prompt modest behavioral change. In the present study, lifestyle factors were not restricted, and participants were asked not to initiate new health-related routines during the trial in order to limit behavioral confounding; no structured diet, exercise, or stress-management counseling was delivered as part of the protocol. Nonetheless, increased health awareness and the structure of repeated study visits may have contributed to symptom improvement in both arms, and such effects cannot be distinguished from placebo responsiveness in this design.

### 4.5. Exploratory Interpretation of the Placebo-Arm Free Testosterone Change

A statistically significant within-arm increase in free testosterone was observed in the placebo group (*p* = 0.0076), whereas the treatment group showed only a non-significant trend (*p* = 0.0727), and the two arms did not differ (*p* = 0.8720). This pattern is unlikely to represent a treatment effect and should be regarded as exploratory. Non-pharmacological explanations apply, including regression to the mean in men selected for low screening testosterone and the substantial within-individual fluctuation of testosterone. Methodological factors are equally important: blood sampling was not time-standardized, the free-testosterone method and assay performance were not specified in the Clinical Study Report, and gonadotropins (LH, FSH), estradiol, and prolactin were not measured. In the absence of repeated, standardized morning measurements and a fuller endocrine panel, these hormonal findings cannot be interpreted as biologically meaningful.

### 4.6. Discrepancy Between Preclinical and Clinical Evidence

These clinical results contrast with previous preclinical findings, including our own study in which the mixed *Elaeagnus multiflora* and *Cynanchum wilfordii* formulation increased testosterone in TM3 Leydig cells and in aging male rats [17], as well as studies reporting androgen-related effects of *Cynanchum wilfordii* in experimental models [18]. Such discrepancies between animal and human studies are common in botanical research. Surgically or chemically induced testosterone deficiency in young animals differs fundamentally from the multifactorial, age-related symptom complex seen in humans [2]. In addition, human participants are aware of being studied, so expectation and other psychological influences—absent in animal models—can shape subjective and possibly physiological outcomes; this difference may itself contribute to the divergence between preclinical and clinical findings.

### 4.7. Consistency with Prior Herbal Medicine Trials in MCS

Our results align with several other herbal medicine trials in MCS. A systematic review of maca (*Lepidium meyenii*) found modest effects on sexual function without consistent testosterone elevation [24], and a randomized trial of unripe black raspberry extract in Korean men with MCS reported symptom improvement without hormonal change [25]. Together, these results suggest that symptom improvement in botanical MCS trials often reflects nonspecific and placebo-related mechanisms rather than a specific androgenic action.

### 4.8. Clinical Meaningfulness of Symptom Improvement

The improvement in both arms (approximately 14–15 AMS points) exceeded the commonly cited minimal clinically important difference of about 10 points [20], indicating that participants experienced perceptible benefit regardless of allocation; this benefit, however, cannot be attributed to EMF_CWW. It should also be noted that the present trial did not evaluate diet, exercise, or other lifestyle interventions and therefore provides no direct evidence for their efficacy. Prior studies have nonetheless reported that weight loss and lifestyle-related factors are associated with changes in testosterone levels in aging men [26], which may be relevant to the comprehensive management of symptomatic aging men. 

### 4.9. Clinical Implications for Non-Hormonal Management

Two cautious implications follow. First, over 12 weeks EMF_CWW was well tolerated, with no treatment-related serious adverse events and no apparent short-term changes in lipids or routine laboratory parameters; this supports short-term tolerability only and does not establish efficacy or long-term safety. On present evidence, EMF_CWW cannot be recommended as an effective alternative to TRT. Second, the comparable improvement across arms indicates that nonspecific elements of care, such as regular follow-up and patient engagement, may accompany symptomatic improvement, although the contribution of any specific intervention cannot be isolated from this trial [4,27].

More broadly, structured clinical attention—regular follow-up, monitoring, and patient engagement—remains valuable in the care of symptomatic aging men, although this design cannot isolate the contribution of any single element [27,28]. Because lifestyle interventions were not evaluated here, no recommendation regarding diet, exercise, or weight management can be drawn from these data; where clinically indicated, however, weight management and lifestyle-related approaches have independent supporting evidence for influencing testosterone-related endocrine profiles and may complement, rather than substitute for, individualized assessment [26].

### 4.10. Implications for Hormonal Assessment in MCS Trials

The hormonal results underscore the difficulty of interpreting single, non-standardized testosterone measurements in this setting. Testosterone varies markedly within individuals, and isolated values may not reflect androgen status [3]. Future trials should use repeated early-morning sampling with a validated free-testosterone method, report assay performance, and include gonadotropins and estradiol to characterize the endocrine response more completely.

### 4.11. Study Limitations

This study has several limitations. First, the enrolled population was not strictly hypogonadal: the total testosterone eligibility ceiling (≤8.59 ng/mL) lies within the normal range and baseline values were low-normal rather than unequivocally low, so the trial is best understood as a study of symptomatic aging men rather than of biochemically defined hypogonadism. Second, the broad age range (45–75 years) introduces heterogeneity in testosterone, SHBG, comorbidity, and symptom burden; exploratory age-stratified analyses were limited by small subgroup sizes and should be regarded as descriptive. Third, the per-protocol set was prespecified as the primary analysis population in the Clinical Study Report; although a conservative intention-to-treat (BOCF) sensitivity analysis yielded the same non-positive conclusion, an intention-to-treat set with prespecified handling of missing data would be preferable as the primary analysis in an efficacy trial. Fourth, hormonal sampling was not time-standardized, the assay was not characterized, and gonadotropins, estradiol, and prolactin were not measured. Finally, the modest sample size and 12-week duration limited power to detect small effects and to assess safety.

### 4.12. Future Research Directions

Future research should prioritize adequately powered, preregistered trials that use intention-to-treat analysis, enrich for participants with rigorously and repeatedly confirmed low morning testosterone, employ standardized repeated morning hormonal measurement with full endocrine characterization, extend follow-up beyond 12 weeks, and provide detailed product characterization in line with reporting standards for herbal interventions. Because the randomized, placebo-controlled design already accounts for nonspecific and placebo-related influences, the priority is not to separate these from treatment effects but to establish, with adequate power and biochemical precision, whether any treatment-specific benefit exists.

## 5. Conclusions

In this 12-week randomized, double-blind, placebo-controlled trial, EMF_CWW did not demonstrate superiority over placebo in improving AMS total score, AMS subdomain scores, ADAM positivity, or hormonal outcomes in symptomatic aging men. Both groups showed substantial symptom improvement, highlighting the potential influence of nonspecific trial effects, placebo responsiveness, regression to the mean, and natural symptom variability in studies of male climacteric syndrome. Hormonal findings should be interpreted cautiously because testosterone sampling was not time-standardized and additional endocrine parameters were not assessed. No treatment-related serious adverse events were observed, and no apparent short-term safety signal emerged during the study period; however, the small sample size and 12-week duration do not allow conclusions regarding long-term safety. Overall, the efficacy of EMF_CWW remains unproven. Larger, preregistered trials using intention-to-treat analyses, rigorously defined biochemical hypogonadism, repeated standardized morning testosterone measurements, longer follow-up, and detailed product characterization are required to clarify whether EMF_CWW has any treatment-specific benefit for male climacteric symptoms.

## Figures and Tables

**Table 1:** Baseline demographic characteristics of participants in the per-protocol population.

Characteristic	Treatment (*n* = 31)	Placebo (*n* = 33)
Age (years)	54.8 ± 7.2	54.5 ± 6.8
Height (cm)	169.5 ± 6.0	171.8 ± 6.8
Weight (kg)	70.2 ± 8.3	72.2 ± 11.0

Values are presented as mean ± SD. Baseline demographic characteristics were summarized descriptively without formal significance testing.

**Table 2:** Changes in AMS, ADAM, and hormonal outcomes during the 12-week study.

Outcome	Group	Baseline	Week 12	Change	95% CI for Change	Within-Group *p*-Value	Between-Group *p*-Value
AMS total	Treatment	45.0 ± 9.5	30.8 ± 11.1	−14.2 ± 12.5	−18.8 to −9.6	<0.001	0.6726
	Placebo	43.7 ± 10.2	28.2 ± 11.0	−15.5 ± 12.3	−19.9 to −11.2	<0.001	—
AMS psychological	Treatment	12.5 ± 3.1	8.1 ± 3.9	−4.5 ± 4.4	−6.1 to −2.8	<0.001	0.8021
	Placebo	12.0 ± 3.8	7.8 ± 3.6	−4.2 ± 4.1	−5.6 to −2.7	<0.001	—
AMS somatic	Treatment	18.4 ± 4.1	12.5 ± 4.9	−5.8 ± 5.6	−7.9 to −3.7	<0.001	0.9930
	Placebo	17.1 ± 4.9	11.3 ± 4.4	−5.8 ± 5.0	−7.6 to −4.1	<0.001	—
AMS sexual	Treatment	14.2 ± 3.8	10.2 ± 4.0	−4.0 ± 4.9	−5.8 to −2.2	<0.001	0.1965
	Placebo	14.6 ± 3.3	9.1 ± 3.5	−5.5 ± 4.8	−7.2 to −3.8	<0.001	—
ADAM positive, *n* (%)	Treatment	29 (93.5%)	15 (48.4%)	—	—	<0.001	0.3264
	Placebo	32 (97.0%)	20 (60.6%)	—	—	<0.001	—
Total testosterone (ng/mL)	Treatment	4.2 ± 1.3	4.0 ± 1.3	−0.2 ± 1.3	−0.7 to 0.3	0.3902	0.3497
	Placebo	4.5 ± 1.2	4.6 ± 1.3	+0.1 ± 1.2	−0.3 to 0.5	0.6783	—
Free testosterone (pg/mL)	Treatment	7.7 ± 2.2	8.6 ± 2.0	+0.8 ± 2.4	−0.1 to 1.7	0.0727	0.8720
	Placebo	7.9 ± 2.6	9.2 ± 2.4	+1.3 ± 2.6	0.4 to 2.2	0.0076	—
SHBG (nmol/L)	Treatment	44.2 ± 13.0	45.9 ± 13.6	+1.7 ± 5.8	−0.4 to 3.8	0.0889	0.3362
	Placebo	54.3 ± 17.8	54.7 ± 18.6	+0.4 ± 7.3	−2.2 to 3.0	0.7889	—

Values are presented as mean ± SD unless otherwise indicated. All data are from the per-protocol set (treatment, *n* = 31; placebo, *n* = 33). Changes were calculated as Week 12 minus baseline. Negative changes in AMS scores indicate symptom improvement. Confidence intervals are presented for mean changes in continuous outcomes only. ADAM positivity is presented as *n* (%). Within-group comparisons were performed using paired *t*-tests for continuous variables and McNemar’s test for ADAM positivity. Between-group comparisons were performed using independent *t*-tests for continuous variables and the chi-square test for ADAM positivity.

**Table 3:** Summary of adverse events during the 12-week intervention.

Category	Treatment Group (*n* = 35)	Placebo Group (*n* = 35)	Total (*n* = 70)	*p*-Value
Participants with ≥1 adverse event, *n* (%)	2 (5.7)	6 (17.1)	8 (11.4)	0.2595
Total number of adverse event episodes, *n*	2	7	9	-
Serious adverse event, *n* (%)	1 (2.9)	0 (0.0)	1 (1.4)	
Non-serious adverse event, *n* (%)	1 (2.9)	6 (17.1)	7 (10.0)	-
Mild, *n* (%)	1 (2.9)	5 (14.3)	6 (8.6)	-
Moderate, *n* (%)	0 (0.0)	1 (2.9)	1 (1.4)	-
Severe, *n* (%)	1 (2.9)	1 (2.9)	2 (2.9)	-
Study product-related adverse event, *n* (%)	0 (0.0)	1 (2.9)	1 (1.4)	
Recovered/resolved without sequelae, *n* (%)	2 (5.7)	6 (17.1)	8 (11.4)	

Values are presented as number (%) unless otherwise indicated. The safety population included all randomized participants who received the study product or placebo. The *p*-value for the overall incidence of adverse events was calculated using Fisher’s exact test. Seriousness, severity, relatedness, and outcome categories are presented descriptively.

## Data Availability

The data presented in this study are available on request from the corresponding author. The data are not publicly available due to institutional policy and ongoing related research.

## Supplementary Materials

The following supporting information can be downloaded at: https://www.mdpi.com/article/10.3390/medicina62071334/s1, Table S1: Summary of Comparative Human Study Schedule; Table S2. Conservative intention-to-treat sensitivity analysis using baseline observation carried forward; Table S3: Changes in AMS score before and after 12 weeks of intake; Table S4: Sub Scale analysis of AMS domain scores (mental, somatic, sexual) and total AMS by age group in the treatment and placebo groups; Table S5: ADAM Ratio After 12 weeks of Intake; Table S6: Hormonal Index Change Before and After 12 Weeks of Intake; Table S7: Change in lipid metabolism markers pre-intake and 12 weeks post-intake; Figure S1: Participant-level changes in individual ADAM questionnaire items from baseline to Week 12; Document S1: HPLC-based characterization and marker information of the EMF_CWW complex extract.
