# Supplementary material for: A Randomized Double-Blind Placebo-Controlled Trial of a Botanical Formulation for Symptom Management in Male Climacteric Syndrome
Source: Medicina (Kaunas). 2026 Jul 10;62(7):1334. doi: 10.3390/medicina62071334 (PMC13414437; doi:10.3390/medicina62071334)
Supplement: Supplementary file 1 [file medicina-62-01334-s001.zip › medicina-4354052-supplementary.pdf]

Supplement Table S1. Summary of Comparative Human Study Schedule.

Category	Screening[a] (Visit 1) Day -14 ~ 0	Baseline (Visit 2) Day 0	Week 6 (Visit 3) Day 42 ± 4	Week 12 (Visit 4) Day 84 ± 4
Informed Consent	●			
Demographics	●			
Medical History	●			
Eligibility Criteria	●	●		
Vital Signs[b]	●	●	●	●
Physical Examination	●	●	●	●
AMS, ADAM Score[c]	●			●
Clinical Lab Tests[d]	●	●		●
Hormonal Tests[e]	●	●		●
Randomization		●		
Product Dispensation		●	●	
Product Return & Adherence			●	●
Medication Confirmation	●	●	●	●
Adverse Events Monitoring		●	●	●

[a]Screening must be conducted within 14 days before Visit 2 (Day 0), and results must be available prior to Visit 2. If results from an approved institution are available within 2 weeks prior to Visit 2, they may be used in place of screening tests.
[b]Blood pressure, Pulse, Temperature
[c] AMS, ADAM: Questionnaire-based assessment.
[d]Clinical Laboratory Tests
·Screening Urinalysis: SG (Specific Gravity), pH, Protein, Glucose, Ketone, Bilirubin, Erythrocyte, Nitrite, Urobilinogen, Leukocyte, Urine Microscopy.
·Screening Hematology: WBC, RBC, Hemoglobin (Hb), Hematocrit (Hct), Platelet, WBC with differential count (Neutrophil, Lymphocyte, Monocyte, Eosinophil, Basophil).
·Screening Blood Chemistry: AST (GOT), ALT (GPT), Creatinine (Cr), eGFR, Glucose.
·Screening and Baseline Specific Tests: PSA, Triglyceride, LDL-cholesterol, HDL-cholesterol, Total Cholesterol (TC).
[e]Hormone Tests
·Screening Test: Total Testosterone
·baseline Test: Free Testosterone, Sex Hormone Binding Globulin(SHBG)

Supplementary Table S2. Conservative intention-to-treat sensitivity analysis using baseline observation carried forward.

Outcome	Treatment group (n = 35), change from baseline	Placebo group (n = 35), change from baseline	Between-group difference	95% CI for between-group difference	Between-group p-value
AMS total score	−12.6 ± 12.6	−14.6 ± 12.5	2.0	−3.9 to 8.0	0.4991
AMS psychological subdomain	−4.0 ± 4.4	−4.0 ± 4.1	0.0	−2.0 to 2.0	0.9798
AMS somatic subdomain	−5.1 ± 5.6	−5.5 ± 5.0	0.3	−2.2 to 2.9	0.7951
AMS sexual subdomain	−3.5 ± 4.8	−5.2 ± 4.8	1.6	−0.7 to 3.9	0.1574
Total testosterone (ng/mL)	−0.2 ± 1.2	0.1 ± 1.2	−0.3	−0.8 to 0.3	0.3450
Free testosterone (pg/mL)	0.7 ± 2.3	1.2 ± 2.5	−0.5	−1.7 to 0.6	0.3723
SHBG (nmol/L)	1.5 ± 5.5	0.4 ± 7.1	1.1	−1.9 to 4.1	0.4584

Values are presented as mean ± SD. Changes were calculated as Week 12 minus baseline. Negative changes in AMS scores indicate symptom improvement. The intention-to-treat population included all randomized participants who received at least one dose of the study product or placebo. Missing Week 12 values were imputed using baseline observation carried forward, assuming no change from baseline. Between-group differences were calculated as treatment minus placebo. Between-group comparisons were performed using independent t-tests.

Supplement Table S3. Changes in AMS score before and after 12 weeks of intake.

Outcome	Treatment Group (n=31)			Placebo Group (n=33)			*p*-value[b]
	Week 0	Week 12	*p*-value[a]	Week 0	Week 12	*p*-value[a]	
AMS total	45.0±9.5	30.8±11.1	<0.0001	43.7±10.2	28.2±11.0	<0.0001	0.6726
AMS psychological	12.5±3.1	8.1±3.9	<0.0001	12.0±3.8	7.8±3.6	<0.0001	0.8021
AMS somatic	18.4±4.1	12.5±4.9	<0.0001	17.1±4.9	11.3±4.4	<0.0001	0.9930
AMS sexual	14.2±3.8	10.2±4.0	<0.0001	14.6±3.3	9.1±3.5	<0.0001	0.1965

Values are presented as mean ± SD

[a]Analyzed by Paired t-test

[b]Analyzed by Independent t-test

Supplement Table S4. Sub Scale analysis of AMS domain scores (mental, somatic, sexual) and total AMS by age group in the treatment and placebo groups.

Age group	Sub Scale	Treatment Mean (SD)	Improvement %	Placebo Mean (SD)	Improvement %
40–49	Psychological	–5.545 (3.698)	39.61%	–4.667 (3.676)	39.44%
	Somatic	–7.000 (4.796)	34.68%	–7.000 (3.908)	39.44%
	Sexual	–4.818 (3.995)	32.71%	–7.083 (3.848)	44.73%
	Total	–17.364 (11.012)	35.50%	–18.750 (8.058)	41.28%
50–59	Psychological	–4.167 (5.323)	32.26%	–4.273 (4.650)	34.06%
	Somatic	–4.833 (7.234)	26.36%	–6.909 (5.262)	38.97%
	Sexual	–4.083 (6.215)	27.68%	–5.545 (4.480)	39.61%
	Total	–13.083 (15.658)	28.44%	–16.727 (13.748)	37.78%
60–69	Psychological	–3.375 (4.173)	34.18%	–3.000 (3.346)	25.64%
	Somatic	–5.625 (4.033)	35.43%	–2.700 (4.855)	17.31%
	Sexual	–2.625 (3.889)	21.00%	–3.400 (5.379)	24.64%
	Total	–11.625 (9.516)	30.39%	–9.100 (13.068)	22.14%

Values are presented as mean change ± SD with corresponding improvement rates (%). Improvement (%) was calculated as (baseline – week 12)/baseline × 100.

Supplement Table S5. ADAM Ratio After 12 weeks of Intake.

ADAM RATIO		Treatment Group (n=31)	Placebo Group (n=33)	*P*-value
Aging Male Syndrome after 12 weeks of taking it	Yes n (%)	15(48.4)	20(60.6)	0.3264
	No n (%)	16(51.6)	13(39.4)	-
Total		31(100.0)	33(100.0)	-

Values are presented as n(%)

Analyzed by Pearson's Chi square-test

Supplement Table S6. Hormonal Index Change Before and After 12 Weeks of Intake.

Hormone Index	Treatment Group (n=31)			Placebo Group (n=33)			*p*-value
	Week 0	Week 12	*p*-value	Week 0	Week 12	*p*-value1)	
Total Testosterone	4.2±1.3	4.0±1.3	0.3902	4.5±1.2	4.6±1.3	0.6783	0.3497
Free Testosterone	7.7±2.2	8.6±2.0	0.0727	7.9±2.6	9.2±2.4	0.0076	0.8720
Sex Hormone Binding Globulin (SHBG)	44.2±13.0	46.2±15.0	0.0889	54.3±17.8	54.7±18.6	0.7889	0.3362

Values are presented as mean ± SD

Analyzed by Paired t-test

Analyzed by Independent t-test

Supplement Table S7. Change in lipid metabolism markers pre-intake and 12 weeks post-intake

Cholesterol	Treatment Group (n =31)			Placebo Group (n = 33)			*p*-value
	0 weeks	12 weeks	*p* -value	0 weeks	12 weeks	*p* -value	
**Total cholesterol**	196.7 ± 42.1	195.8 ± 35.7	0.8246	191.3 ± 26.8	193.9 ± 23.1	0.4744	0.5076
**Triglyceride**	183.0 ± 99.5	176.1 ± 90.7	0.6826	178.7 ± 173 .6	148.1 ± 128. 7	0.3431	0.5148
**HDL-cholesterol**	51.5 ± 12.4	50.0 ± 11.5	0.2973	56.1 ± 14.4	56.1 ± 14.6	0.9538	0.4174
**LDL-cholesterol**	119.6 ± 36.2	120.5 ± 31.2	0.7726	112.6 ± 25.5	114.9 ± 22.4	0.4831	0.7657

Values are presented as mean ± SD

Analyzed by Paired t-test

Analyzed by Independent t-test

a

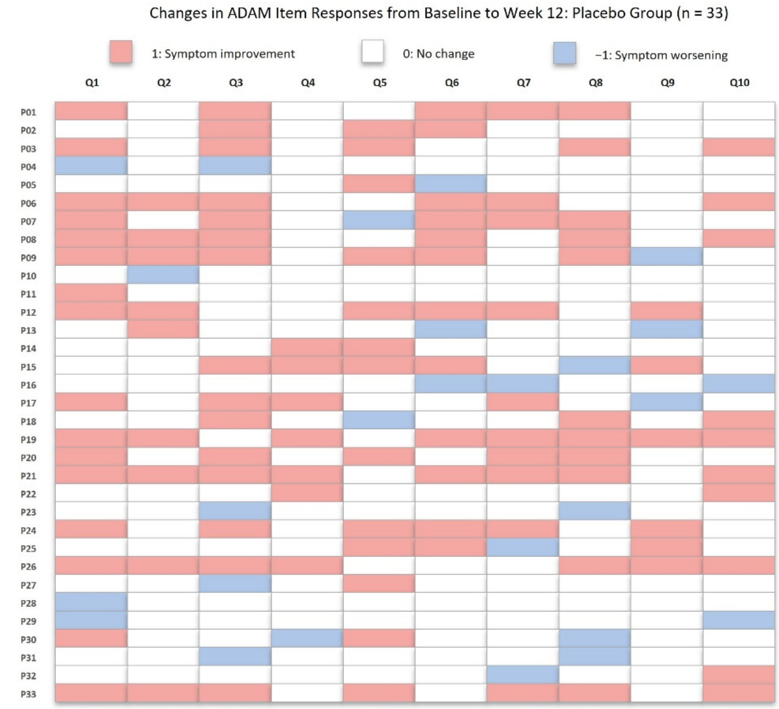

b

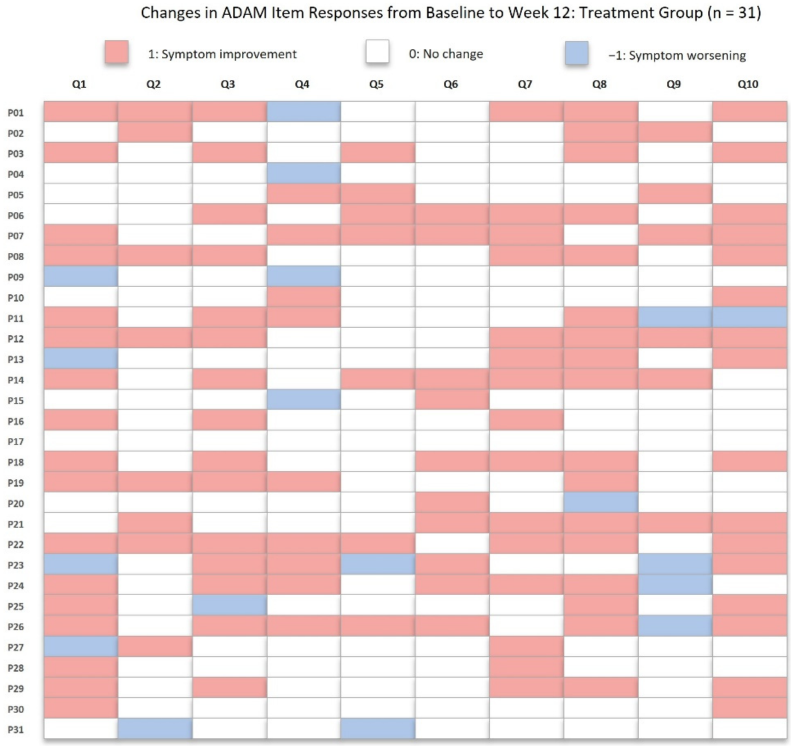

Supplement Figure S1. Participant-level changes in individual ADAM questionnaire items from baseline to Week 12.

(a) Placebo group (n = 33). (b) Treatment group (n = 31). Each column corresponds to an ADAM questionnaire item (Q1–Q10), and each row represents one participant. Red indicates symptom improvement (+1), defined as a change from a positive response at baseline to a negative response at Week 12; white indicates no change (0); and blue indicates symptom worsening (−1), defined as a change from a negative response at baseline to a positive response at Week 12. Q1, decreased libido; Q2, lack of energy; Q3, decreased strength or endurance; Q4, loss of height; Q5, decreased enjoyment of life; Q6, sadness or grumpiness; Q7, weaker erections; Q8, reduced ability to engage in sports; Q9, falling asleep after dinner; Q10, deterioration in work performance.
