# Supplementary material for: A Randomized Double-Blind Placebo-Controlled Trial of a Botanical Formulation for Symptom Management in Male Climacteric Syndrome
Source: Medicina (Kaunas). 2026 Jul 10;62(7):1334. doi: 10.3390/medicina62071334 (PMC13414437; doi:10.3390/medicina62071334)
Supplement: Supplementary file 1 [file medicina-62-01334-s001.zip › medicina-4354052-supplementary Document S1.pdf]

HPLC-Based Characterization and Standardization of the EMF_CWW Complex Extract

In response to the Editor's request, we provide the chromatographic characterization and marker-based standardization data for the herbal complex extract (EMF_CWW) administered in the clinical trial. EMF_CWW is a freeze-dried water-extract mixture of *Elaeagnus multiflora* Thunb. fruit (EMF) and *Cynanchum wilfordii* (Maxim.) Hemsl. root (*Cynanchi Wilfordii Radix*; CWW), combined at a CWW:EMF ratio of 7:3 (w/w). The marker compounds and the corresponding HPLC profiles are described below.

S1. Preparation of the herbal extracts

S1.1. EMF water extract. Dried *Elaeagnus multiflora* fruit (25 kg) was rinsed with distilled water and extracted with 10 volumes of distilled water (250 L) at 100°C for 4 h (single extraction). The extract was filtered through 400-mesh filter cloth, concentrated to 15 °Brix, and freeze-dried to obtain 3.1 kg of EMF water extract (extraction yield, 12.4%).

S1.2. CWW water extract. Dried *Cynanchi Wilfordii* Radix (20 kg) was processed under matched conditions [10 volumes of distilled water (200 L), 100°C, 4 h, single extraction; 400-mesh filtration; concentration to 15 °Brix; freeze-drying], yielding 2.3 kg of CWW water extract (extraction yield, 11.5%). The extract was obtained as a white powder free of off-odor and off taste.

S1.3. EMF_CWW complex extract. The two freeze-dried extracts were combined at a CWW:EMF ratio of 7:3 (w/w) to constitute the EMF_CWW complex extract used in the clinical trial.

Table S1. Extraction conditions and yields of the individual extracts.

Parameter	EMF (*E. multiflora* fruit)	CWW (*C. wilfordii* radix)
**Starting material**	25 kg	20 kg
**Extraction solvent**	Distilled water, 10 vol (250 L)	Distilled water, 10 vol (200 L)
**Temperature / time**	100°C / 4 h (×1)	100°C / 4 h (×1)
**Filtration**	400 mesh	400 mesh
**Concentration**	15 °Brix	15 °Brix
**Drying**	Freeze-drying	Freeze-drying
**Extract obtained / yield**	3.1 kg (12.4%)	2.3 kg (11.5%)
**Marker (content in extract)**	Phenolic acid-type (≥0.1%)	4′-Hydroxyacetophenone (≥0.5%)

S2. Marker compounds

The CWW marker was 4′-hydroxyacetophenone (4-acetylphenol; molecular formula $C_8H_8O_2$; molecular weight 136.15 g/mol; reference standard, Sigma-Aldrich cat. no. 278564; CAS 99-93-4). The CWW water extract contained ≥0.5% (w/w) 4′-hydroxyacetophenone.

The EMF marker was a phenolic acid-type compound, monitored as the characteristic peak of the EMF water extract; the EMF water extract contained ≥0.1% (w/w) of this marker.

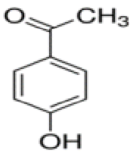

Figure S1. Chemical structure of 4′-hydroxyacetophenone, the CWW marker compound.

S3. HPLC results

S3.1. EMF water extract (Figure 2). The chromatogram of the EMF water extract showed the phenolic acid-type marker peak at a retention time of approximately 28.1 min.

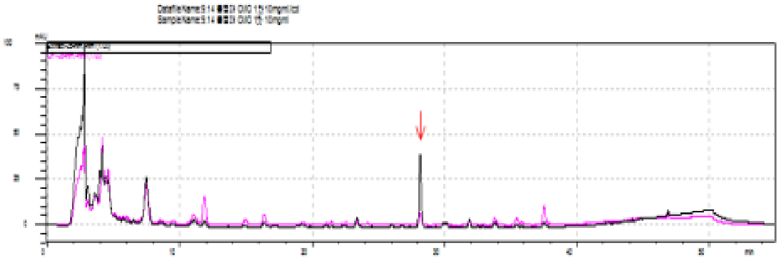

Figure S2. HPLC chromatogram of the *Elaeagnus multiflora* fruit (EMF) water extract. The phenolic acid-type marker peak is indicated by the arrow (retention time 28.1 min). Sample, 10 mg/mL

S3.2. CWW water extract (Figure 3). The chromatogram of the CWW water extract showed a prominent peak for 4′-hydroxyacetophenone at a retention time of approximately 15.3 min.

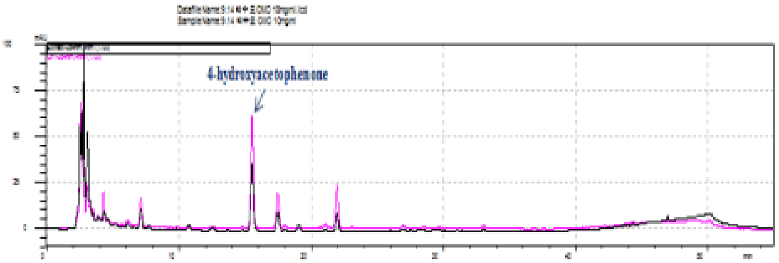

Figure S3. HPLC chromatogram of the *Cynanchi Wilfordii* Radix (CWW) water extract. The 4′-hydroxyacetophenone peak is indicated (retention time 15.3 min). Sample, 10 mg/mL

S3.3. EMF_CWW complex extract, 7:3 (Figure 4). In the complex extract analyzed at a CWW:EMF ratio of 7:3 (w/w), both markers were detected: 4′-hydroxyacetophenone (CWW marker) at a retention time of approximately 15.3 min and the phenolic acid-type compound (EMF marker) at approximately 28.1 min. The two marker peaks were baseline-resolved and did not co-elute. These observations indicate that the chromatographic conditions allow the simultaneous monitoring of both markers and are therefore suitable for the standardization and quality control of the EMF_CWW complex extract.

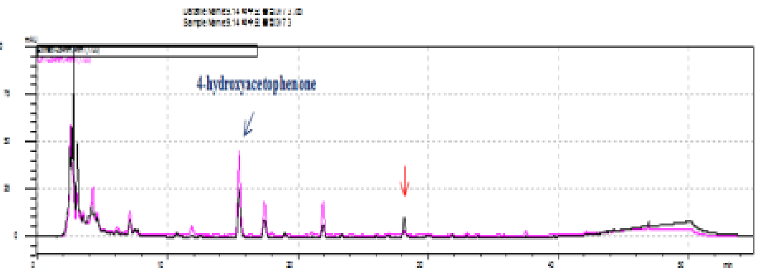

Figure S4. HPLC chromatogram of the EMF_CWW complex extract (CWW:EMF = 7:3, w/w). The CWW marker 4′-hydroxyacetophenone (retention time 15.3 min) and the EMF phenolic acid-type marker (retention time 28.1 min) are baseline-resolved.
